# Technical Feasibility of Extraction of Freshwater from Produced Water with Combined Forward Osmosis and Nanofiltration

**DOI:** 10.3390/membranes14050107

**Published:** 2024-05-03

**Authors:** Madina Mohamed, Marco Tagliabue, Alberto Tiraferri

**Affiliations:** 1 Department of Environment, Land and Infrastructure Engineering, Politecnico di Torino, Corso Duca degli Abruzzi, 24, 10129 Torino, Italy; madina.mohamed@polito.it; 2 Eni S.p.A., Research and Development, Via F. Maritano, 26, 20097 San Donato M.se, Italy

**Keywords:** produced water, forward osmosis, nanofiltration, draw solution

## Abstract

This study assesses the technical feasibility of a forward-osmosis-based system for concentrating produced water and extracting freshwater. Forward osmosis was combined with nanofiltration, the latter system used to restore the initial osmotic pressure of the diluted draw solutions while concurrently obtaining the final freshwater product. Three draw solutions, namely, MgCl_2_, NaCl, and C_3_H_5_NaO_2_, were initially tested against a synthetic water mimicking a pretreated produced water effluent having an osmotic pressure equal to 16.3 bar. MgCl_2_ was thus selected for high-recovery experiments. Different combinations of draw solution osmotic pressure (30, 40, 60, 80, and 120) and draw-to-feed initial volume ratios (1, 1.6, and 2.2) were tested at the laboratory scale, achieving recovery rates between roughly 35% and 70% and water fluxes between 4 and 8 L m^−2^h^−1^. One-dimensional, system-wide simulations deploying the analytical FO water flux equation were utilized to validate the experiments, investigate co-current and counter-current configurations, and understand the system potential. The diluted draw solutions were then transferred to nanofiltration to regenerate their original osmotic pressure. There, the highest observed rejection was 96.6% with an average flux of 21 L m^−2^h^−1^, when running the system to achieve 100% relative recovery.

## 1. Introduction

Petroleum and other underground energy resources still play a substantial role in the modern economy [1]. While society is transitioning toward the use of alternative resources [2], almost all industries currently depend on petroleum products at one stage or another, e.g., fuel production or petrochemical manufacturing. Other than the final high-value products, oil and gas extraction and processing produce a considerable number of low-value ones. A chief example is produced water (PW) [3,4,5,6,7]. Produced water is a complex brine brought to the surface along with hydrocarbons and minor condensates, and mixed with water streams that originated in the separation processes of hydrocarbons. Globally, 248.4 million barrels of PW are generated from oil production per day, from both onshore and offshore wells [7,8,9,10].

Two common handling methods of PW are disposal and reuse [6,11,12]. The latter exploits PW as a potential freshwater resource [6], according to United Nations Sustainable Development Goals 6 and 12 [https://sdgs.un.org/goals (accessed on 29 April 2024)]. However, a suitable treatment of PW is required, depending on the specific application, e.g., process, potable, sanitary, and crops irrigation. The applications can range from reinjection into oil or gas reservoirs for enhanced oil recovery to, potentially, high-end uses in the agricultural and civil sectors [13]. PW requires intensive treatment to be used outside the energy sector, such as for irrigation purposes, even when these do not involve crops or products for human consumption [12]. Therefore, it is essential that we develop a series of strategies for PW treatment and for the extraction of water to comply with the necessary standards [5,14,15].

Various methods are being employed or developed aimed at the treatment of PW [9,16]. Among those or in the framework of certain management schemes, membrane separation processes may provide scalability and a suitable separation performance [8,11,15,17,18,19,20,21,22,23,24,25,26,27,28,29,30,31,32,33]. The complexity of the PW composition presents a challenging task in selecting suitable unit operations for its treatment. Microfiltration or ultrafiltration using ceramic or polymeric membranes, though effective in removing suspended materials, demand post-treatment to enhance the quality of the treated effluent, e.g., denser membrane separation steps [10,20,25,30,34]. Reverse osmosis is impractical for many PWs due to their high salinity and composition, since typical PWs contain non-soluble oil/organics and chemicals that would render intensive pre-treatment necessary prior to the RO step. These characteristics would limit the productivity, the recovery rate, and/or the permeate quality in RO [4,35,36]. Membrane distillation offers opportunities for treating highly saline solutions, but with high energy expenses [26,37]. Numerous researchers have employed a hybrid approach that integrates membrane filtration with other processes to improve the effluent quality and system efficiency. As representative examples, Purnima et al. reported that microfiltration followed by biological treatment removed 99% of organic matter rather than using a single operation unit [10]. Riley et al. introduced a hybrid membrane biosystem that was able to remove over 99% of organic matter and 94% of TDS from PW [15].

Forward osmosis is an osmotically driven membrane technology that has been suggested as a valid tool for the treatment of PW under certain circumstances, especially combined with other treatment units [19,21,22,27,28,38,39,40,41,42,43,44,45,46,47,48,49,50]. The driving force of the water transport in an FO process is owed to the inherent osmotic pressure difference across the membrane. A draw solution (DS) facilitates the freshwater passage from the feed solution (FS), which has a comparatively lower salinity and ultimately lower osmotic pressure than the DS. Indeed, in order to maintain the driving force and ensure the continuous, feasible, operation of the FO system, the diluted draw solution must be reconcentrated after its dilution, a step that simultaneously allows water extraction [51]. Such a second system step depends on the nature and the concentration of the draw agent to be reconcentrated and represents the energy-intensive unit operation within the water recovery scheme. An interesting aspect of this approach is that it provides a dual barrier for contaminant removal, whereby one step, namely, the FO unit treatment, requires little energy [52]. Moreover, while not widely implemented, FO is presently a mature technology, with reliable providers able to provide installation on a medium and large scale [53].

A number of past studies have investigated the implementation of FO in the treatment of PW, but the technical feasibility of this strategy has not been fully understood [54]. Nawaza et al. investigated the efficiency of a combined FO–membrane-distillation system for the treatment of four different PW streams sourced from a single industrial site [43]. The water–oil stream served as the DS for FO with measured water fluxes ranging from 8.3 L m^−2^h^−1^ (LMH) to 26.8 LMH in the FO step, and 5.6 LMH to 11.1 LMH in the membrane distillation step. Stable fluxes in both FO and membrane distillation were achieved, with minimal fouling and adequate product water quality. One prominent issue that hinders the investigated system is that membrane distillation is not yet a mature technique. In another study conducted by Chen et al., the effectiveness of two FO membranes, a commercial cellulose triacetate membrane and a custom-made thin film composite membrane, was evaluated for treating shale gas drilling flow-back fluids [55]. Using a synthetic brine as the DS, the TFC membrane provided higher fluxes but was also affected by fouling problems, compared to the cellulose triacetate membrane. Another relevant study conducted by Minier-Matar et al. assessed the use of FO for the concentration of PW aimed at its disposal by deep well injection. Two main operating parameters were investigated in this study, namely, the DS concentration (from 35 to 175 g/L NaCl) and temperature. The experimental findings showed that a 50% volume reduction in PW was achieved with a stable flux of 12 LMH using 1 M NaCl as the draw solution. Organic matter from the FS did not transfer considerably into the diluted DS, thus minimizing its discharge into the environment. However, neither study evaluated DS reconcentration [42].

The aim of the current study is to provide further insight into the technical feasibility of a system comprising FO and nanofiltration (NF) for the extraction of freshwater from PW and for the regeneration of the DS. The discussion starts with data obtained in laboratory-scale experiments and centered around the water fluxes and water quality observed under high-recovery experiments. For simplicity, a synthetic PW is used, based on a real stream. The experimental data are then deployed to validate the analytical, system-scale modeling of both units, namely, FO and NF, thus allowing an investigation of conditions that would also fall outside those investigated experimentally. The scope of this work is partly limited by the use of a synthetic FS and by the scale of the experiments, but the results and analyses provide a benchmark for the assessment of the technical feasibility, or lack thereof, of an FO-based system in the management of typical PW of streams with similar salinity and composition.

## 2. Materials and Methods

### 2.1. Membranes and Chemicals

Thin-film composite polyamide membranes were used for the FO experiments. Following the protocol reported previously by Tiraferri et al. [56], membrane characterization was performed on a number of separate samples (>4) using a laboratory-scale FO system to determine the active layer water permeance, *A*, the MgCl_2_ permeability coefficient, *B*, and the support layer structural parameter, *S*. MgCl_2_ was used as the draw solute of choice for such characterization and deionized water as the feed solution. The values obtained were 2.74 ± 0.50 LMH, 0.07 ± 0.25 LMH, and 427 ± 19 µm, respectively. The membrane performance was robust, and the values obtained with different samples fit within an adequate range. For the NF tests, flat-sheet DuPont NF90 membranes were purchased from Oltremare (Fano, Italy).

The solutions were prepared using magnesium chloride hexahydrate (MgCl_2_∙6H_2_O, purity ≥ 99.8%), calcium chloride (purity ≥ 99.8%), sodium acetate, boric acid, and sodium chloride (NaCl) (purity ≥ 99.8%), purchased from Carlo Erba (Cornaredo, Italy), sodium sulfate (purity ≥ 99%) purchased from Chem-lab (Zedelgem, Belgium), and potassium chloride (KCl) (purity ≥ 99%) purchased from Sigma Aldrich (St. Louis, MO, USA). Acetic acid (purity ≥ 99.8) was used for adjusting the feed solution pH and was purchased from Carlo Erba. Three different draw agents were preliminarily assessed, namely, sodium chloride, magnesium chloride, and sodium propanoate (purity ≥ 99.8%) purchased from Carlo Erba. DI water from a Milli-Q (Merck, Italy) system was used in all preparations.

### 2.2. Feed Solution Characteristics

The feed solution used in this study was synthesized to simulate the composition of pre-treated PW, e.g., de-oiled, filtrated, and degassed. The composition details were obtained from the operator’s own analyses, including concentrations of specific constituents, pH, and resulting electric conductivity. Table S1 of Supplementary Materials presents the main characteristics of the feed solution.

### 2.3. Lab-Scale Membrane Filtration Setups

The FO lab-scale plant was purchased from Sterlitech Corporation (Kent, WA, USA). It is composed of two reservoirs for the FS and the DS, respectively, with a capacity of 7 L each. It includes a membrane cell (acrylic Sepa cross-flow cell, Sterlitech, Auburn, WA, USA) with dimensions 146 mm long, 94.5 mm wide, and 1.5 mm deep. The active area of the membrane sample was 140 cm^2^. The membrane was used in the so-called “FO mode”, with the active layer facing the FS and the support layer facing the DS. The system comprises two variable speed gear pumps (Cole-Parmer, Vernon Hills, IL, USA), one for each stream loop, and a data acquisition system. The DS tank was placed on a digital scale balance (Kern Instruments, Balingen, Germany) connected to a computer to record the change of volume during the experiment, and, hence, calculate the water flux. For all the experiments, the crossflow rate was 1.8 L/min for both the feed and the draw sides, while the temperature of both streams was maintained at 21 ± 1 °C by submerging in the two tanks stainless-steel heat exchanger coils connected to an external chiller. Both the FS and the DS streams were recirculated back to the respective reservoirs during the experiments.

A crossflow NF system was used for conducting all draw agent regeneration experiments. The unit is composed of a high-pressure pump (Hydra-cell pump, Wanner Engineering, Inc., Minneapolis, MN, USA), feeding reservoir, flat-sheet membrane housing cell (stainless steel Sepa cross-flow cell, Sterlitech, Auburn, WA, USA), temperature control, and data acquisition system. The housing cell comprises a channel with length 147 mm, width 97 mm, and height 1.9 mm. The membrane active area was thus 140 cm^2^. A floating disc rotameter was utilized to monitor the crossflow rate. Both the crossflow and operating pressure were controlled and adjusted by means of a bypass valve and a back-pressure regulator (Swagelok, Solon, OH, USA), while a computer-interfaced balance automatically monitored and recorded the permeate flow rate every 1 min. The retentate streams were recirculated back to the respective reservoir, while the permeate stream was collected in an external tank placed on a computer-interfaced balance. The temperature in the retentate tank was controlled via a recirculating chiller (Model MC 1200, Lauda, Lauda-Königshofen, Germany) accompanied with a stainless-steel coil heat exchanger coil submerged in the tank to maintain the temperature at 21 ± 1 °C.

### 2.4. FO and NF Tests

In preliminary tests, various draw agents were assessed based on their water flux performance in low-recovery tests. Specifically, the synthetic PW (Table S1 of Supplementary Materials) was used as FS while measurements of steady-state water fluxes were conducted over five steps, characterized by different values of the DS osmotic pressure. Following the selection of the most suitable draw agent, namely, MgCl_2_, high-recovery FO tests were performed. A minimum of two replicates were conducted for each test to guarantee experimental repeatability.

The main experimental design of this study is represented in Figure S1 (Supplementary Materials) and consisted of a high-recovery FO test conducted on the synthetic produced water, followed by a high-recovery NF test conducted with the diluted draw solution from the previous FO step. The high-recovery FO tests were conducted as follows: 2.5 L of synthetic PW was used as FS. The initial MgCl_2_ DS osmotic pressure and/or volume were varied for each experiment. The tested osmotic pressure values were 30, 40, 60, 80, and 120 bar, while the volumes were 2.5 L, 4 L, and 5.5 L, resulting in DS to FS initial volume ratios of 1, 1.6, or 2.2; see Table S2 of Supplementary Materials for a summary of the experimental conditions. Different DS-to-FS-initial-volume ratios in lab-scale tests represent different DS-to-FS-influent-flow-rate ratios in real-scale scenarios. The relationship between osmotic pressure and draw agent concentration was obtained using “OLI Studio” software (https://www.olisystems.com/software/oli-studio/, OLI Systems Inc., Parsippany, NJ, USA). The FS and DS were recirculated back to their respective reservoirs, during which the former was concentrated, and the latter was diluted. The water flux was measured every 10 min until approximately 65–70% of the initial FS volume permeated from the feed to the draw side, or until the flux went to near zero, whichever condition was reached first. Following each FO test, the diluted DS was used as solution to be reconcentrated in the NF tests. In the high-recovery NF tests, a 100% relative water recovery rate was targeted; i.e., the same volume of water that had previously diluted the DS was made to permeate the NF membrane. At the beginning of each FO test, an aliquot of the initial FS and DS solutions was sampled. At the end of each FO test, samples were collected from the concentrated FS and the diluted DS, while, at the end of each NF test, the regenerated DS and the permeated products were sampled.

### 2.5. Water Characterization

Elemental analysis on the liquid samples diluted with aqueous nitric acid (HNO_3_, Merck, superpure grade) was conducted by inductively coupled plasma optical emission spectrometry (ICP-OES) using a Thermo Fisher ICAP 6000 instrument. The calibration was performed from standards certified by the external calibration method (VWR). Total carbon (TC) was also quantified and used to quantity organic carbon (TOC) with a Shimadzu TOC V CPH analyzer equipped with a Shimadzu NDIR detector. Dissolved anions were quantified by ion chromatography with a Thermo Scientific Dionex ICS 6000 ion chromatograph equipped with an eluent generator (potassium hydroxide, KOH) Thermo Scientific Dionex EGC III, a precolumn Thermo Scientific Dionex Ionpac AG11-HC, a Thermo Scientific Dionex Ionpac AS11 column, and a conductivity meter.

### 2.6. FO and NF Water Flux Simulations

Simulations of the approximate FO water flux as a function of recovery rate or membrane area were performed by application of the FO water flux equation [51,57]:

$$J_{w}^{FO}=A\left\{\frac{\pi_{D}\cdot \exp\left(-\frac{J_{w}S}{D}\right)-\pi_{F}\cdot \exp\left(-\frac{J_{w}}{k^{FO}}\right)}{1-\frac{B}{J_{w}}\left[\exp\left(\frac{J_{w}}{k^{FO}}\right)-\exp\left(-\frac{J_{w}S}{D}\right)\right]}\right\}$$

where *D* is the diffusion coefficient of the draw solute in water, *π*_D_ and *π*_F_ represent the draw and feed bulk osmotic pressures, and *k^FO^* is the mass transfer coefficient at the active layer–solution interface, which is a function of the hydrodynamics of the feed channel and was fixed at the value of 40 LMH, which represents the average value computed for the FO cell utilized in this work. The same value for *k^FO^* was applied to simulate the experimental results in co-current mode and to compute the FO productivity in predictive simulations under both co-current and counter-current configurations.

Simulations of the experimental NF water flux as a function of recovery rate were performed by application of the NF water flux equation:

$$J_{w}^{NF}=A\left[\Delta P-\sigma \cdot \Delta \pi \cdot \exp\left(\frac{J_{w}}{k^{NF}}\right)\right]$$

where *σ* is the reflection coefficient, assumed to be equal to 1 for simplicity, Δ*P* is the applied transmembrane pressure, and *k^NF^* is the mass transfer coefficient at the active layer–solution interface, which is a function of the hydrodynamics of the feed channel and was fixed at the value of 100 LMH, which represents the average value computed for the NF cell utilized in this work.

In cross-flow systems, whether FO or NF, the water flux would vary along the membrane module due to the change in driving force. Therefore, the water flux was calculated through the discretization of the theoretical length of the membrane module by numerical iteration of the equations above, to obtain the amount of water passing across the membrane in each discrete portion of the system. For each portion, the concentrations and the resulting osmotic pressure were thus recalculated through mass and flow balances, to obtain the full profiles along the system.

## 3. Results and Discussion

### 3.1. Choice of the Draw Solute and Operating Conditions

A series of preliminary FO experiments were conducted with the synthetic PW as FS for the selection of the draw agent and of the operating conditions for the high-recovery experiments. Three draw solutions were assessed, namely, MgCl_2_, NaCl, and C_3_H_5_NaO_2_. The results of the steady-state water flux as a function of the DS concentration are presented in Figure S2 (Supplementary Materials). The results of the simulations run by applying the analytical FO water flux model are also displayed for NaCl and MgCl_2_ as the DS, and plotted as continuous lines in Figure S2. These calculations were performed without any additional fitting parameters other than the previously determined membrane transport parameters, i.e., *A*, *B*, *S*, and the known cross-flow channel mass transfer coefficient, *k*, which was equal to 40 LMH for the FO cell and the cross-flow conditions deployed in the tests. The highest fluxes were observed with NaCl, likely due to its high diffusion coefficient, which reduced the dilutive internal concentration polarization compared to other compounds. Overall, fluxes between 6 and 9 LMH were observed at a DS bulk osmotic pressure of 42 bar, and fluxes between 12 and 18 LMH at a DS bulk osmotic pressure of roughly 95 bar. Note that these preliminary tests also provided experimental validation of the FS bulk osmotic pressure, equivalent to the value of the DS bulk osmotic pressure at which the modeled fluxes went to zero. This value was equal to 16.3 bar. Despite MgCl_2_ providing lower fluxes than the other two draw agents, flux values were sufficiently close to those obtained with the other agents to make other considerations important. Specifically, MgCl_2_ is a universally available and relatively inexpensive salt, which may be recovered using tight nanofiltration membranes, whereas other compounds would necessarily require either highly dense membranes or other less established techniques, e.g., evaporative ones, for recovery. The hypothesis of this work, which also underlies several other research investigations of forward osmosis systems is the following: while nanofiltration on a divalent salt does not necessarily entail a lower energy need than reverse osmosis for the purpose of DS reconcentration, a combination of higher fluxes and rejections may be advantageous in terms of the system compactness and efficacy of draw agent recovery. The following chapters set forth to discuss the results based on this initial hypothesis.

### 3.2. Observed Productivity of the FO Step and Validation of the Water Flux Simulations

**Figure 1:**
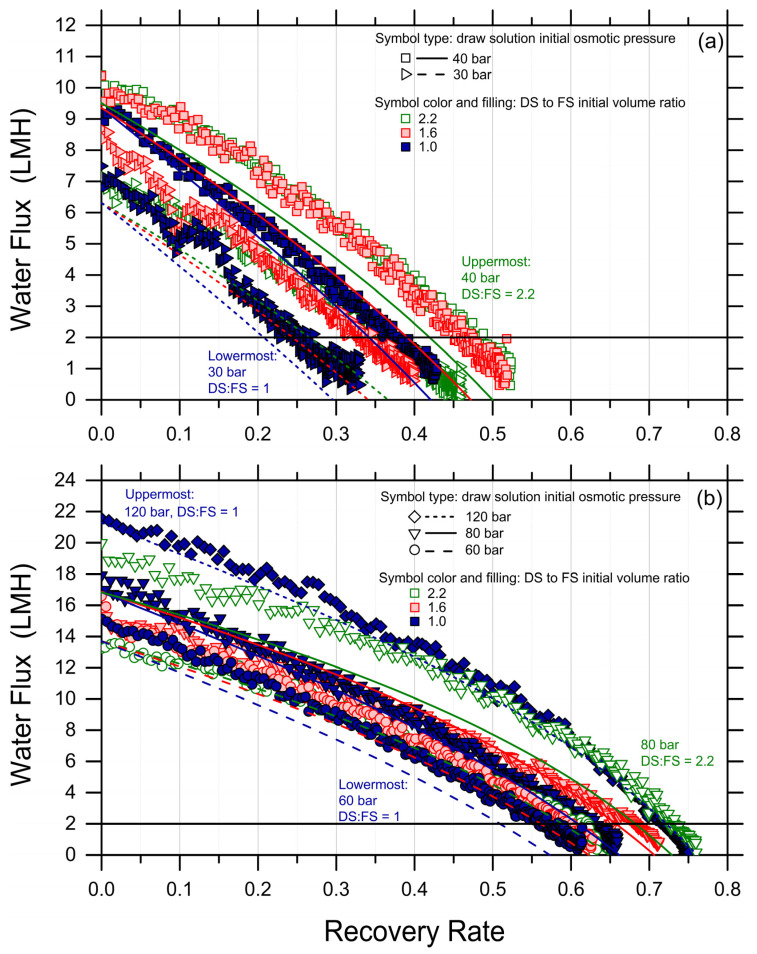
Water fluxes (data points) measured and (lines) simulated in forward osmosis as a function of water recovery rate. The initial draw solution bulk osmotic pressures were 30 bar ((**a**); rightward triangles for the experimental data, continuous lines for the simulated data); 40 bar ((**a**); squares, dash lines); 60 bar ((**b**); circles, dash lines); 80 bar ((**b**); downward triangles, continuous lines); and 120 bar ((**b**); diamonds, dotted lines). The draw-to-feed initial volume ratios were: 1 (solid blue experimental data, blue lines); 1.6 (shaded red experimental data, red lines); and 2.2 (empty green experimental data, green lines). Tests were conducted with a synthetic produced water pretreated effluent as FS (Table S1, Supplementary Materials), at 21 ± 1 °C, with a membrane active area of 23 cm^2^, and cross-flow rates of 1.8 L/min in both FS and DS channels. The horizontal black lines at 2 LMH correspond to a hypothetical lower bound of process feasibility in terms of productivity.

High-recovery tests were conducted with the synthetic PW as FS and MgCl_2_ solutions as DS using different combinations of DS initial osmotic pressure and DS-to-FS-initial-volume ratios; see Table S2 (Supplementary Materials). Figure 1 reports the observed water fluxes as a function of the recovery rate. The continuous lines represent the fluxes predicted with a forward simulation that combines volume and osmotic pressure balances for each high-recovery experimental condition. Therefore, they are not the result of data fitting, but independent results obtained by applying FO water and solute analytical equations for each time step as a function of the recovery rate. The results are akin to those that would be obtained along a cross-flow membrane module as a function of the recovery rate prevailing in each point of the module. A perfect membrane rejection was assumed for the simulations since no substantial difference in the flux prediction was observed when the rejection was assumed to be as low as 97%. Note that the experimental and simulation results were divided into two graphs with different scales of the ordinate axis, simply for a better visualization of the otherwise large amount of data.

As expected, both the experimental and simulated water fluxes showed a decline as a function of the recovery rate, attributed to the reduction in the driving force as water moved from the feed side to the draw side. Average water fluxes spanned from roughly 4 to 10 LMH; see Table 1. Moreover, according to theoretical expectations, the higher the initial DS osmotic pressure and the higher the DS-to-FS-initial-volume ratio, the larger both the measured fluxes and the achieved recovery rates, generally. To assess the feasibility of the technique, the achieved recovery rate values were estimated for each condition at the point where the water flux profile crossed the 2 LMH horizontal black line in Figure 1. This choice was made to be conservative and because lower fluxes may not be considered technically feasible in real operation. Given this assumption, the highest recovery rate of approximately 73% was achieved under two conditions: (I) an initial DS osmotic pressure of 120 bar and a draw-to-feed initial volume ratio of 1 (solid, blue diamonds in Figure 1b), and (II) an initial DS osmotic pressure of 80 bar and a draw-to-feed initial volume ratio of 2.2 (empty, green, downward triangles in Figure 1b). The lowest recovery of 25% was observed in the test employing the lowest DS initial osmotic pressure, i.e., 30 bar, and the lowest DS-to-FS-initial-volume ratio of 1. See also Table 1 and Figure S3 (Supplementary Materials) for a graphical summary of the achieved recovery rate values.

**Figure 2:**
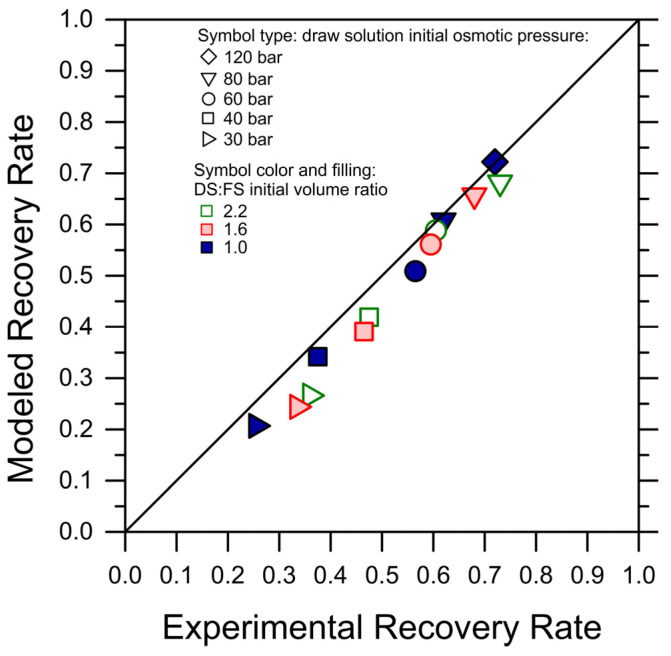
Simulated recovery rates vs. experimentally observed recovery rates for each tested condition of DS initial osmotic pressure and DS-to-FS-initial-volume ratio. For this purpose, the recovery rates were retrieved as the water flux reached the lowest value of 2 LMH. The color and symbol codes of the data points are consistent with Figure 1. The 45-degree line is also plotted.

While the simulations predicted the initial water fluxes and the trends in an adequate fashion, in a few cases, they underestimated the flux values and the final recovery rate, with a discrepancy compared to the experiments of up to 20%. This discrepancy may be partly due to experimental variation, to a systematic underestimation of the membrane transport performance, or to other phenomena not captured by the simple FO transport equations, but it suggests that fouling did not occur considerably during the tests and that forward simulations may be used as a conservative means to predict the recovery rate achieved under conditions not explored experimentally. Indeed, when plotting the experimental recovery rate against the simulated recovery rates, the data sit remarkably close to the 45-degree line; see Figure 2.

### 3.3. Predicted Productivity of the FO Step

**Figure 3:**
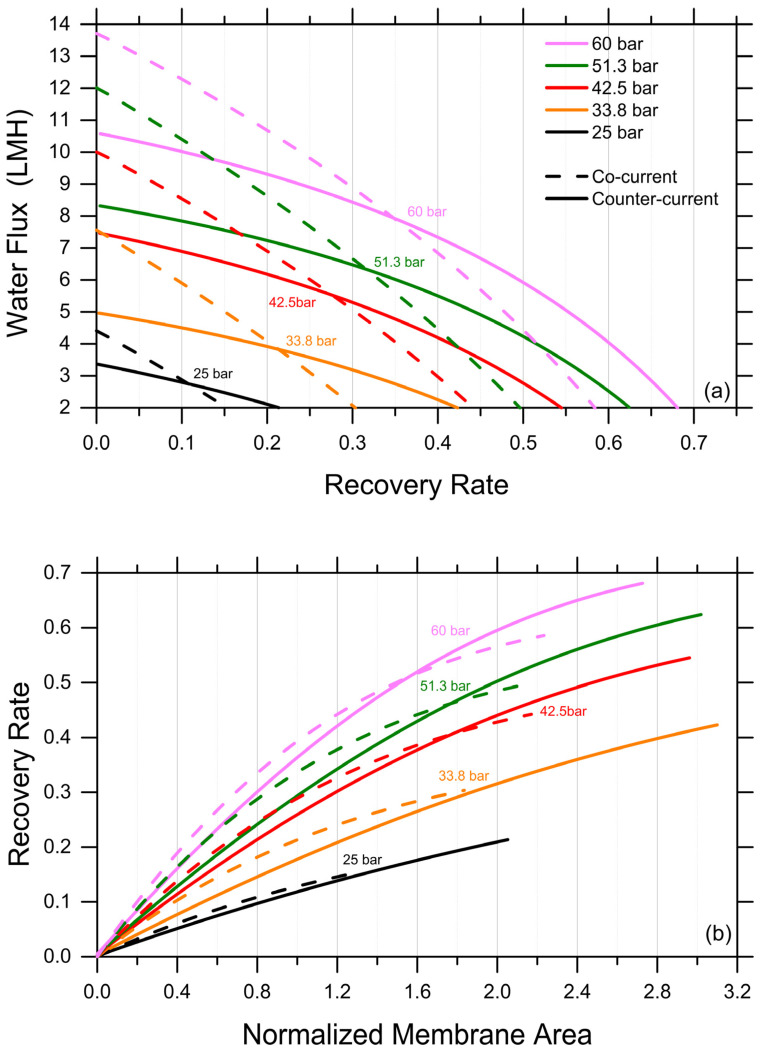
Productivity of an FO system simulated under co-current vs. counter-current configurations. (**a**) Water fluxes as a function of recovery rate. (**b**) Recovery rate as a function of normalized membrane area. Representative results for the case of MgCl_2_ as draw agent, a DS-to-FS-influent-flow-rate ratio of 2, and stopping the simulations at water flux values equal to 2 LMH.

Discussed herein are selected results obtained by one-dimensional, system-wide simulations deploying the analytical FO water flux equation, which was discussed above as adequate and conservative in predicting recovery rates under various scenarios. A first exploration provides insights into the impact of co-current and counter-current configurations. It is important to note that the experiments described above implicitly represent a co-current configuration, as the increasingly concentrated FS are put in contact through the membrane with the increasingly diluted DS. The simulations were stopped when the water fluxes reached a lower limit of 2 LMH. As expected, counter-current configurations were predicted to be characterized by more uniform fluxes as a function of the recovery rate and to consistently yield higher recovery rates; see Figure 3a, which presents the results obtained with a DS-to-FS-influent-flow-rate ratio of 2 and MgCl_2_ as the draw agent. Specifically, the predicted, achievable recovery rate in the counter-current configuration was between 40% higher, for an influent DS osmotic pressure of 25 bar, and 15% higher, for an influent DS osmotic pressure of 60 bar, than that associated with a co-current configuration, reaching a value of roughly 67.5% for the latter DS influent osmotic pressure. DS osmotic pressure values larger than 60 bar were considered unfeasible from a practical point of view. As illustrated in Figure 3b, a counter-current configuration does not necessarily provide larger recovery rates when the same membrane area is available in the system, but it allows for the exploiting of a considerably larger membrane area with fluxes larger than 2 LMH compared to a co-current configuration, ultimately resulting in a substantially higher recovery rate for a reasonable investment in terms of the membrane area.

**Figure 4:**
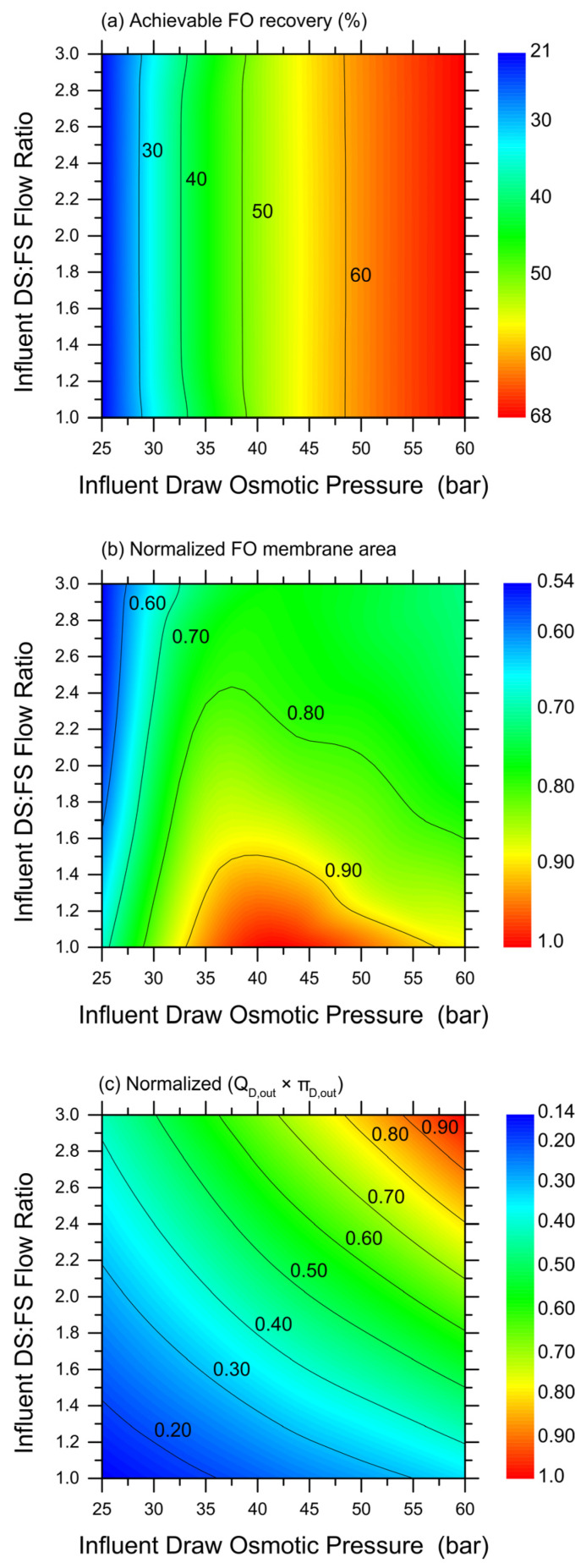
Simulation-based predictions of FO system behavior and productivity in co-current configuration, as a function of DS influent osmotic pressure and DS-to-FS-influent-flow-rate ratio. Color maps of: (**a**) achievable recovery rate in the FO step; (**b**) relative required FO membrane area (normalized by the highest value in the map); and (**c**) empirical parameter representing the product between effluent DS flow rate (*Q*_D,out_) and effluent DS osmotic pressure (*π*_D,out_). Blue and red colors represent low and high values of the investigated parameter, respectively. Simulations were run with MgCl_2_ as draw agent and until a water flux equal to 2 LMH was calculated.

While the selected results plotted in Figure 3 provide a first understanding of the potential range of FO system productivity, Figure 4 presents a more complete set of predictions based on simulations. In particular, a wide range of simulations were performed under the more advantageous counter-current configuration, by varying the DS (MgCl_2_) influent osmotic pressure and the DS-to-FS-influent-volume ratio. Then, the obtained, achievable recovery rates (up until a water flux of 2 LMH) were simply interpolated to create the color maps illustrated in Figure 4a–c, which would allow the estimation of the (Figure 4a) achievable recovery rate and (Figure 4b) relative exploitable FO membrane area under a wide range of conditions. Note that, because they are the result of the interpolation of discrete results obtained with analytical simulations, these maps are not fully rigorous or analytical. The results suggest that the DS-to-FS-influent-flow-rate ratio would not have an impact on the achievable recovery rate, and the DS influent osmotic pressure governs instead this system response. However, the FO membrane area required to achieve such recovery rates would be lower at high DS:FS influent flow rate ratios, and this mechanism is expected due to the lower DS dilution along the membrane modules. In particular, the map related to the relative FO membrane area illustrates a non-trivial trend, with a maximum for a medium range of the DS influent osmotic pressure and low DS-to-FS-influent-flow-rate ratios. Therefore, an optimization seems possible to maximize the recovery rate and minimize the required FO membrane area.

Indeed, it would be simple to increase the recovery rate and reduce the required FO membrane area by working at a larger DS influent flow rate and/or a larger DS influent osmotic pressure. However, such conditions may render the subsequent DS regeneration step unfeasible. To obtain a qualitative assessment of the overall complexity of the nanofiltrations step aimed at extracting 100% of the freshwater previously recovered in the FO step and to completely regenerate the DS at the influent values of the osmotic pressure, the fully empirical parameter depicted in Figure 4c was calculated. This parameter is the product between the flow rate of the DS exiting the FO step, which coincides with the feed solution entering the NF step, and the DS osmotic pressure exiting the FO step. In fact, as the two variables increase, larger areas and lower fluxes would be associated with the NF system, with a near equal contribution from the two; see Figure 4c. Overall, the combined plots in Figure 4 provide insight into the most suitable combination of FO conditions that may be technically feasible while translating into a lower full system complexity. Just as an example, working with a DS influent osmotic pressure of about 52.5 bar and at a DS-to-FS-influent-flow ratio of 1.4 seems to allow the achievement of a recovery rate of approximately 62% with average water fluxes of 6.0 LMH, reasonably low FO membrane areas, and limiting the complexity of the NF step, the latter being the subject of the next chapter.

### 3.4. Draw Solution Regeneration and Freshwater Extraction with Nanofiltration

**Figure 5:**
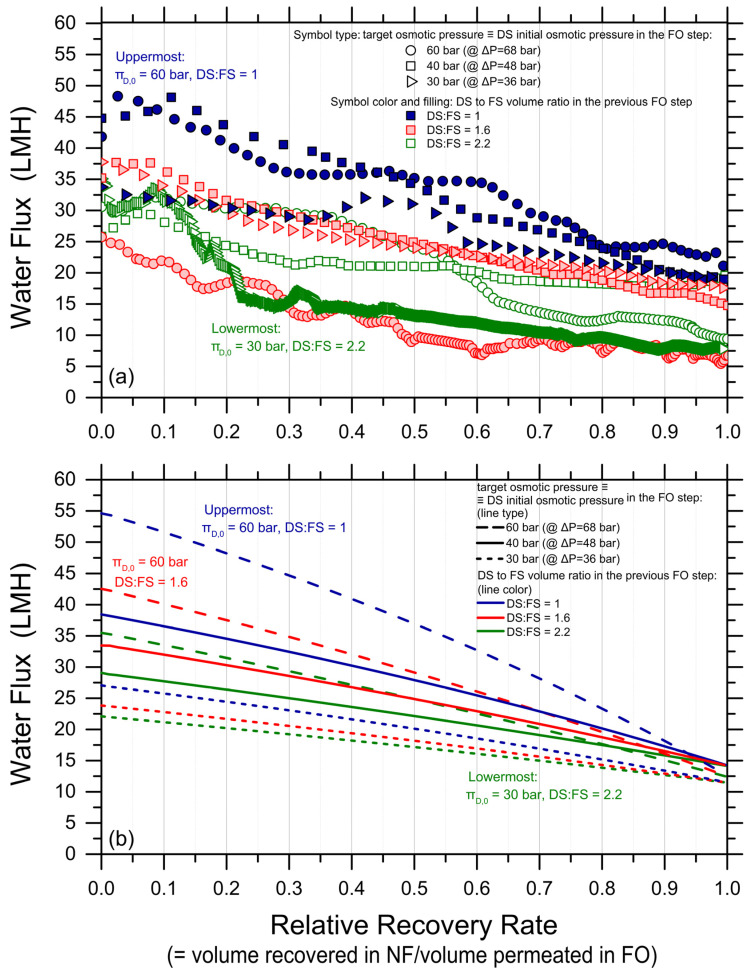
Water fluxes in nanofiltration as a function of relative recovery rate; i.e., the relative recovery rate is calculated as the ratio of the volume of water recovered in nanofiltration (NF) to the volume previously permeated in forward osmosis (FO). (**a**) Experimental data and (**b**) results of simulation using the NF analytical water flux equation taking into account concentration polarization by applying a feed channel mass transfer coefficient of Commercial NF90 membranes were used (Dupont). The applied pressure was in the range of 36–68 bar depending on the initial osmotic pressure of the synthesized draw solution. In (**a**), circles, squares, and triangles refer to DS that needed to be regenerated to 60 bar, 40 bar, and 30 bar, respectively. Blue, red, and green colors refer to diluted DS obtained in previous FO experiments with DS to FS initial volume ratios equal to 1, 1.6, and 2.2, respectively.

Water fluxes measured in the nanofiltration tests are reported in Figure 5. The diluted draw solutions from nine FO experiments were regenerated in NF, corresponding to DS initial osmotic pressures in FO (coinciding with target osmotic pressure upon NF reconcentration) of 30, 40, and 60 bar and draw-to-feed initial volume ratios of 1, 1.6, and 2.2 (each pressure tested at three different ratios). The applied pressures in the NF experiments were 36, 48, and 68 bar, respectively, for the three target osmotic pressure values. In this work, no obvious damage was observed for any of the NF membranes. However, note that commercial NF membrane modules have a maximum pressure rating that may be lower than the pressure values applied in this study. As expected, due to the increasingly high osmotic pressure of the concentrate solutions along the tests, Figure 5a shows that the water flux decreased with the increasing relative recovery rate. Water fluxes spanned from roughly 10 to 33 LMH, substantially higher than those observed in the FO step. A 100% relative recovery rate was obtained in all the NF tests. Note that the trends observed in NF were not as neat as those measured in FO. Moreover, the experimental data were predicted only in a broad way by the forward simulations performed using the NF analytical water flux equation; see Figure 5b and Figure S4 (Supplementary Materials). The discrepancies may be due to an instability of the NF system and/or of the NF membrane at high applied pressure, which ranged between 36 and 68 bar. 

### 3.5. Quality of the Product Water

An analysis of the composition of all streams entering and exiting the FO and NF units was conducted. The majority of cations and metals exhibited a medium to high retention by the FO membrane. Specifically, the observed rejection was 97.5% for Na, 66.3% for B, 99.3% for Ca, and 94.8% for K, on average. Additionally, the acetate and sulfate concentration in the draw side were under the detection limit, indicating a near-total retention by the membrane. No substantial increase in magnesium or chloride was observed in the feed solution, suggesting a limited reverse solute flux occurring during the tests. When considering the nanofiltration step, the average retention of Mg and Cl by the NF membrane was 93% and 81.9%, respectively. Table 2 shows the characteristics of the final product water obtained through the nanofiltration treatment of the diluted DS coming from the high-recovery experiments. The TOC content was low, corroborating that the FO-NF double barrier prevented the passage of acetate. However, the total dissolved solids (TDSs) concentration of the final product water, coinciding with the nanofiltration permeate stream, was in the range of mildly brackish water and also contained a certain amount of sodium. These results suggest that the FO-NF double barrier allows the non-negligible passage of monovalent ions and, more importantly, that nanofiltration membranes may not be sufficiently dense to retain the DS at a sufficient rate. Similar conclusions may be drawn by analyzing the data in terms of MgCl_2_ regeneration. The so-called “MgCl_2_ system rejection” presented in Table 1 was calculated as the draw solute concentration in the final volume of the extracted freshwater divided by the MgCl_2_ concentration in the initial DS used for the relative FO experiment. The highest system rejection was 96.6%. Note that, in most tests, the overall FO-NF system rejection lay between 90 and 96%, but, in two cases, it was considerably lower than expectations. The low rejection values may be due to experimental issues, including a non-optimal membrane sample and/or errors in the water analysis, and should not be considered reflective of the expected behavior of a perfectly functioning system in the opinion of the authors. Overall, the results suggest that, while a complete recovery of the freshwater could be achieved, a non-negligible fraction of the draw agent would be lost throughout the system, mostly due to the incomplete rejection in the NF step, but also partly due to scaling and through losses as a reverse solute flux in FO. Practically, in order to maintain the target osmotic pressure, 5–10% of the initial agent would need to be consistently added to the recirculated draw stream. As discussed also by previous studies, such losses may, in some cases, represent an important limitation, resulting in the impaired technical and economic feasibility of the combined system [51,58,59,60]. In this sense, RO membranes may be necessary for the correct regeneration of MgCl_2_ or other similar solutes, which would lower the productivity of this second system step and require larger membrane areas than those that would be associated with a nanofiltration unit [61,62].

## 4. Conclusions

The aim of the current study is to provide insight into the technical feasibility of a system comprising forward osmosis and nanofiltration for the extraction of freshwater from a synthetic produced water with an osmotic pressure of 16.3 bar, representative of a mix from upstream fossil fuel extraction activities and of minor condensates and water flows originated in the separation processes of hydrocarbons. High-recovery FO tests were performed using MgCl_2_ as the draw agent at varying values of the draw solution osmotic pressure and draw-to-feed volume ratios. The results of the simulations based on the FO transport equations were thus validated, and then applied to predict the recovery rates achievable under a wider range of operating conditions. High-recovery NF tests and simulations were finally performed to describe the ability in extracting freshwater and regenerating the DS. Note that this study has some limitations, among which are the use of a synthetic feed solution, and the limited duration of the experiments that did not allow an appraisal of the fouling behavior. That being said, the tests conducted at high recovery, supported by validated simulations, provide important insight into the technical feasibility of the system for this specific treatment case, but also for a wide variety of applications targeting feed solutions in the same osmotic pressure range.

A combination of forward osmosis and nanofiltration would allow the production of brackish water in the low-salinity range, starting from a produced water with an osmotic pressure of roughly 16 bar (salinity of approximately 20 g/L) and at recovery rates between 55 and 65% (if working under the counter-current configuration in the FO step). Based on both experimental and modeling results, the overall system would have important drawbacks. One drawback is the inability to produce freshwater at a salinity lower than 500 mg/L. Another limitation, already pointed out by previous studies, would be the low productivity in the FO step, with average fluxes in the order of 6 to 8 LMH with the influent MgCl_2_ draw solution characterized by an osmotic pressure of around 50 bar. This high osmotic pressure, combined with the imperfect rejection rate of the solute in nanofiltration, implies the need for the substantial replenishment of the draw agent at each recirculation (5–10% in mass). The system may thus require the use of highly dense RO membranes in the second step, even with a 2:1 draw agent such as MgCl_2_. With RO membranes and at a similar or only marginally higher applied pressure with respect to those associated with NF membranes, a higher draw agent rejection would be achieved at the expense of some loss in the water flux, but still maintaining average productivities that are likely substantially higher than those associated with the first FO step. Overall, while the first (FO) step seems to be the limiting factor in terms of the water productivity [63], the second (NF or RO) step seems, instead, to be the limiting factor in terms of the ability of saving the draw agent and producing a high-quality final water product, when the membranes are not highly dense. Attempting a rational evaluation of the technical feasibility of the FO-based system under similar conditions, this study suggests, along with several recent investigations on the feasibility of forward osmosis, that this technique is likely suitable for applications entailing the dilution or the concentration of high-value solutions and/or when an inexpensive draw agent or draw solution is widely available. Applications aiming at extracting high-quality freshwater from streams of medium or high salinity may be feasible at medium to high costs, with the materials (e.g., membrane, and draw agents) that are available today. Any detrimental effects, such as fouling or membrane deterioration, or other requirements, such as membrane cleaning, chemical additions, and pre- or post-treatment, none of which were investigated in this study, would add potential further complexity to the system. The complexity and variability of the real produced water could add further issues. Note that the scope of this study does not include any economic or environmental considerations, which would be necessary to make a final, informed decision on the applicability of the technique.

## Figures and Tables

**Table 1:** Summary of water flux and recovery results in the FO and NF tests.

DS Initial Osmotic Pressure (bar)	DS-to-FS-Initial-Volume Ratio	Average Experimental FO Water Flux ^a^ (LMH)	Experimental Recovery Rate @ 2 LMH	Average Experimental NF Water Flux ^b^ (LMH)	MgCl_2_ System Rejection
30	1	4.2	25%	26.3	94
30	1.6	4.4	34%	24.0	79.5
30	2.2	4.2	38%	13.3	96.3
40	1	4.9	34%	30.5	90.5
40	1.6	5.3	38%	23.6	93
40	2.2	5.5	41%	21.0	96.6
60	1	6.9	50%	32.3	87.5
60	1.6	7.3	54%	10.9	74.8
60	2.2	6.7	58%	18.6	95.5
80	1	7.9	62%	n.a.	n.a.
80	1.6	7.3	63%	n.a.	n.a.
80	2.2	9.6	65%	n.a.	n.a.
120	1	10.2	73%	n.a.	n.a.

^a^ Between the initial value and the value of 2 LMH, ^b^ Applied feed hydraulic pressure were: 36 bar, 48 bar, and 68 bar, for target final concentrated DS with osmotic pressure of 30 bar, 40 bar, and 60 bar, respectively, n.a.: not available (NF experiment not performed).

**Table 2:** Composition of the final product water from combined FO and NF filtration tests.

DS Initial Osmotic Pressure (bar)	DS-to-FS-Initial-Volume Ratio	TOC (ppm)	Na (ppm)	Mg (ppm)	Cl^−^ (ppm)	B (ppm)	TDS (ppm)
30	1	2.3	137	344	882	<0.3	~1360
30	1.6	4.2	168	1560	3300	<0.3	~5030
30	2.2	1.3	245	288	800	<0.3	~1330
40	1	1.5	93	773	1420	<0.3	~2290
40	1.6	1.7	160	669	1520	<0.3	~2350
40	2.2	1.1	94	723	1490	<0.3	~2310
60	1	3.9	210	1270	2630	<0.3	~4110
60	1.6	2.4	134	1300	2700	<0.3	~4130
60	2.2	1.2	153	480	1150	<0.3	~1780

## Data Availability

The data will be made available upon request from the corresponding authors.

## Supplementary Materials

The following supporting information can be downloaded at https://www.mdpi.com/article/10.3390/membranes14050107/s1. Table S1: Characteristics of the synthetic produced water used as feed solution; Table S2: Initial osmotic pressures of the DS and draw-to-feed initial volume ratios utilized in high-recovery FO experiments; Figure S1: Scheme representing the experimental design of this work; Figure S2: Fluxes measured in forward osmosis low-recovery tests as a function of draw solution osmotic pressure, for three different draw agents; Figure S3: Recovery rates observed in high-recovery FO tests as a function of DS-to-FS-initial-volume ratio and DS initial osmotic pressure; Figure S4: Simulated average NF water fluxes vs. experimentally observed average fluxes.
