# Supplementary material for: Technical Feasibility of Extraction of Freshwater from Produced Water with Combined Forward Osmosis and Nanofiltration
Source: Membranes (Basel). 2024 May 3;14(5):107. doi: 10.3390/membranes14050107 (PMC11123107; doi:10.3390/membranes14050107)
Supplement: Supplementary file 1 [file membranes-14-00107-s001.zip › membranes-2919942-supplementary.pdf]

Supporting Information for

Technical Feasibility of Extraction of Freshwater from Produced Water with Combined Forward Osmosis and Nanofiltration

Madina Mohamed [1], Marco Tagliabue [2,*], and Alberto Tiraferri [1,*]

[1]*Department of Environment, Land and Infrastructure Engineering, Politecnico di Torino, Corso Duca degli Abruzzi, 24, 10129 Torino, Italy*

[2]*Eni S.p.A., Research and Development, Via F. Maritano, 26, I-20097 San Donato M.se, Italy*

*Corresponding authors:

M.T.: marco.tagliabue@eni.com

A.T.: alberto.tiraferri@polito.it

Table S1 Characteristics of the synthetic produced water used as feed solution

Parameter	Produced water
pH	7.8
Conductivity (μS/cm)	61741
Na^{+} (ppm)	2700
Ca^{2+} (ppm)	1500
K^{+} (ppm)	500
Mg^{2+} (ppm)	500
Acetic acid (ppm)	4700
Cl^{-} (ppm)	8500
SO_4^{2-} (ppm)	1300
$B(OH)_3$ (ppm)	60

Table S2. Initial osmotic pressures of the DS and draw-to-feed initial volume ratios utilized in high-recovery FO experiments.

Initial osmotic pressure (bar)	***DS to FS initial volume ratio***		
30	1	1.6	2.2
40	1	1.6	2.2
60	1	1.6	2.2
80	1	1.6	2.2
120	1		

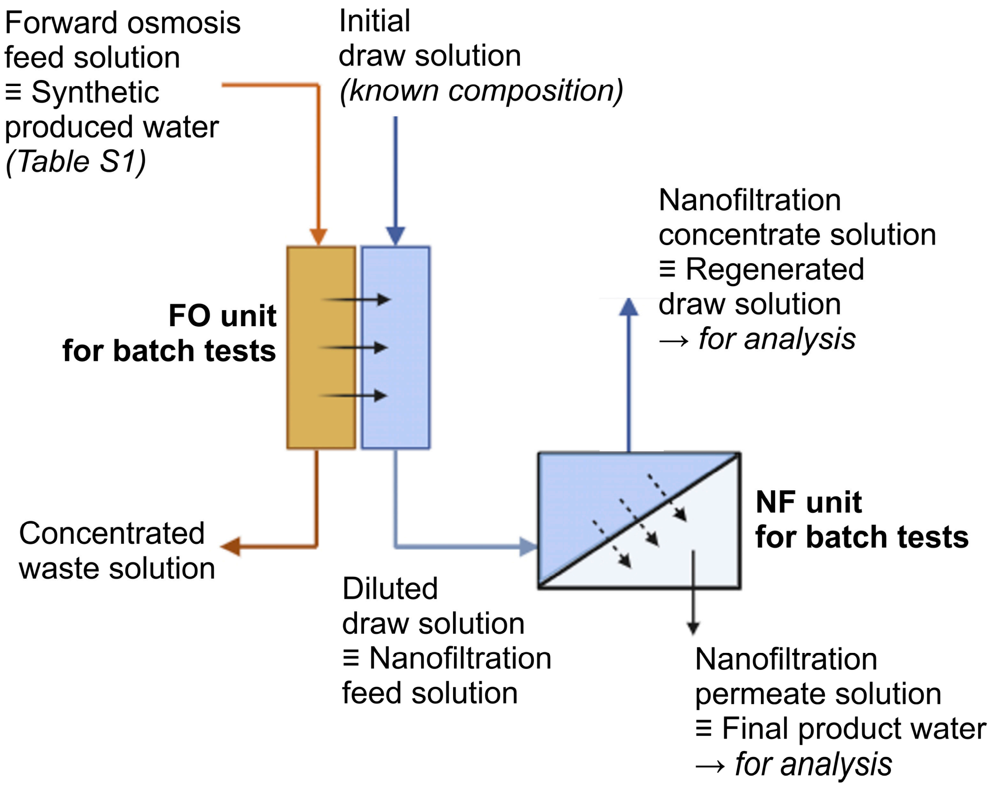

Figure S1. Scheme representing the experimental design of this work. A synthetic produced water is first treated in an FO lab-scale unit to extract freshwater using a draw solution of known composition. The diluted draw solution is then treated in an NF lab-scale unit to (i) produce a freshwater product and to (ii) regenerate the draw solution, both the latter solutions being analyzed to assess the feasibility of the treatment scheme.

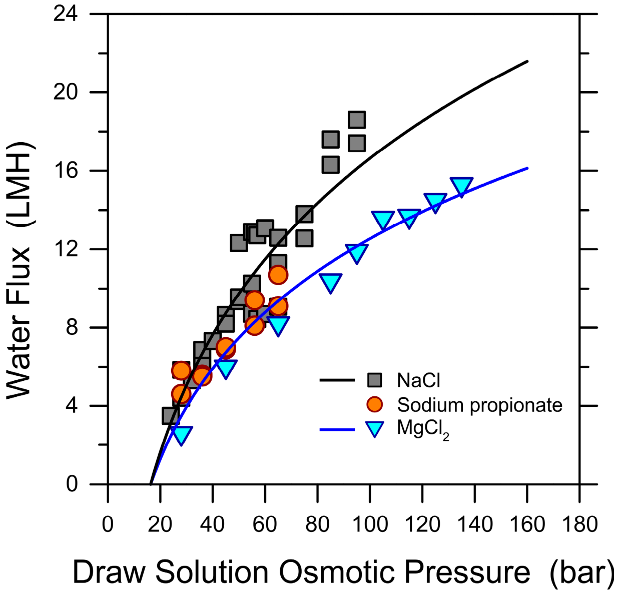

Figure S2. Fluxes measured in forward osmosis low-recovery tests as a function of draw solution osmotic pressure, for three different draw agents. The feed solution was synthetic produced water. Grey, orange, and blue points represent experimental data obtained with NaCl, $C_3H_5NaO_2$, or $MgCl_2$ as draw agent, respectively. The black and blue continuous lines are the results of the FO analytical water flux model, calculated using the membrane characteristics, a channel mass transfer coefficient of 40 LMH, and the diffusion coefficient of NaCl, and $MgCl_2$, respectively, at 21 °C.

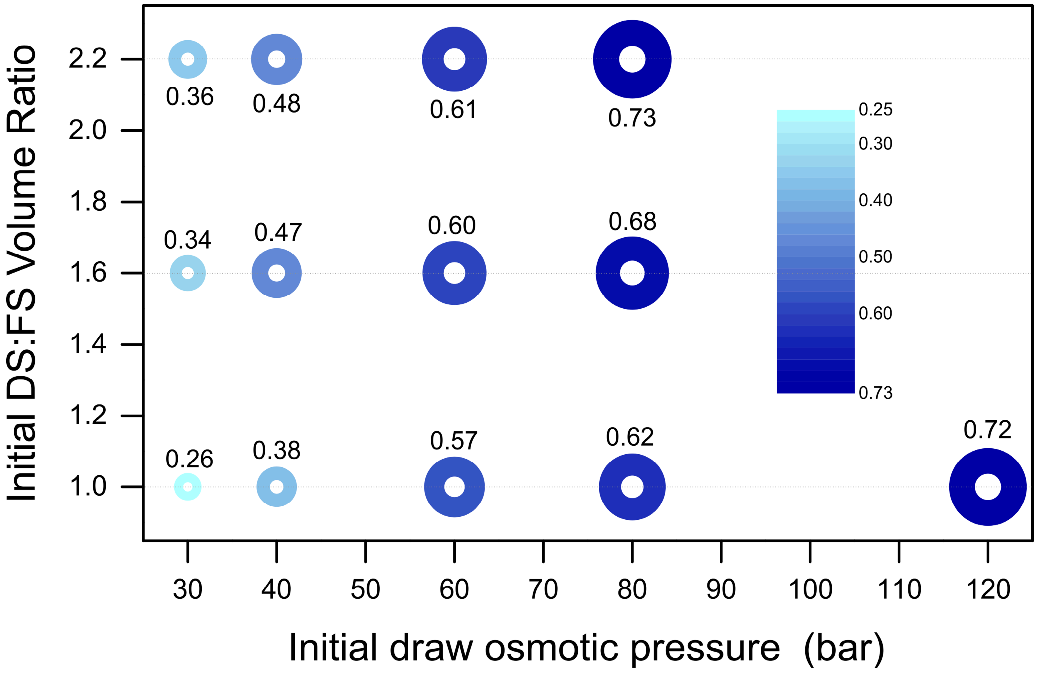

Figure S3. Recovery rates observed in high-recovery FO tests as a function of DS to FS initial volume ratio and DS initial osmotic pressure. Light and dark blue colors represent low and high values of the achieved recovery, respectively. Experiments were run with $MgCl_2$ as draw agent.

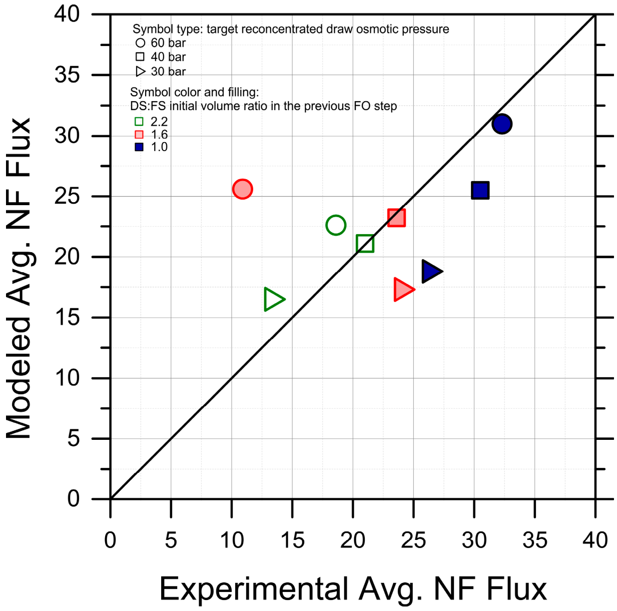

Figure S4. Simulated average NF water fluxes vs. experimentally observed average fluxes. Simulations performed for each tested condition of DS target osmotic pressure and DS to FS initial volume ratio employed in the previous FO step used to dilute the DS. The color and symbol codes of the data points are consistent with Figure1 in the main manuscript. The 45-degree line is also plotted.
